# Electrical Strength and Physicochemical Performances of HTV Silicone Rubber under Salt-Fog Environment with DC Energized

**DOI:** 10.3390/polym12020324

**Published:** 2020-02-04

**Authors:** Zhijin Zhang, Tian Liang, Chen Li, Xingliang Jiang, Jian Wu, Bin Wu

**Affiliations:** 1 State Key Laboratory of Power Transmission Equipment & System Security and New Technology, Chongqing University, Chongqing 400044, China; liangtian@cqu.edu.cn (T.L.); 20115109@cqu.edu.cn (C.L.); xljiang@cqu.edu.cn (X.J.); 2 State Grid Chongqing Electric Power Company, Gaoxin District, Chongqing 400039, China; hzhwujian@126.com (J.W.); cqdlwubin@163.com (B.W.)

**Keywords:** silicone rubber, salt-fog, DC electrical strength, hydrophobicity, SEM, FTIR, dielectric parameter

## Abstract

In recent years, the performances of rubber composite insulators, which operate in the coastal foggy regions, have attracted researchers’ concern because of the observation of their degradation. In this paper, salt-fog experiments with DC test voltage of high-temperature vulcanized (HTV) silicone rubber (SR) have been conducted. The electrical strength and material performances of samples with salt-fog treatment were focused on. The DC flashover voltage, hydrophobicity, scanning electron microscopy (SEM), Fourier transform infrared spectroscopy (FTIR), and dielectric parameter were investigated. It was found that the samples’ performances deteriorated after salt-fog treatment. The DC flashover voltage of HTV SR decreased in the salt-fog environment. The hydrophobicity of the material deteriorated and the static contact angle (CA) became small. Under the action of electric and thermal stress, the surface of samples after salt-fog treatment became rough and porous. The absorption peak of the hydrophobic groups decreased, indicating that the molecular chain of SR material was broken, and the filler was consumed, bringing down the arc resistance of the sample. The absorption of moisture further led to insulation performance loss and then reduced the electrical strength of the material. Degradation of physicochemical properties will eventually lead to a decline in electrical strength.

## 1. Introduction

Rubber composite insulators using high-temperature vulcanized (HTV) silicone rubber (SR) as the sheaths and sheds material are widely applied in the power systems of China in recent decades for their many advantages, such as excellent antipollution flashover performance, light weight, convenient installation, and especially the hydrophobicity and hydrophobicity transfer. They provide better electrical and physicochemical performances compared with the traditional porcelain and glass insulators [1,2,3,4].

However, silicone rubber sheds suffer from various kinds of external environmental stresses in practice, such as pollution, ultraviolet radiation, heat, salt-fog, etc. The performance of silicone rubber composite insulators will be degraded under multiple stresses, which may cause severe damage to the electrical transmission system. For example, the long chain structure of silicon rubber can be broken to short ones in the environment of high-energy rays, which will reduce the hydrophobicity of silicone rubber, resulting in a reduction of flashover voltage [5,6]. The absorption of moisture increases the dielectric constant and dielectric loss factor (tanδ), which leads to local heating on the insulator and poor insulation performance, decreasing the breakdown filed strength of HTV SR [7,8]. Hydrophobic surfaces can still be destroyed in heavy contamination, thus forming dry bands before the recovery of hydrophobicity [9]. The electric field also affects the performance of the silicone rubber insulator. The water drop on the surface of composite insulators can be elongated, forced by the voltage stress, making the static contact angle decrease [10]. In turn, the electric field will be enhanced by water drops, especially at the interface of the water drop and the insulator surface, the corona caused by which plays a negative role in the long-term operating of composite insulators [11]. In a word, under the influence of various electrical stresses and environmental stress, silicone rubber material will inevitably age, which has become a widespread concern in scientific research and operation departments [12].

In recent years, China has constructed several EHV and UHV power transmission projects, and some others are about to start. In this condition, long-distance transmission lines will travel through complex climate regions. In southern China, a large number of composite insulators have been installed in the coastal areas with a salt-fog environment, and we have observed that there have been some performance changes of running composite insulators [13]. There has been a decrease of flashover voltage of insulators in the salt-fog environment—salt-fog has an independent effect on this downward trend [14]. It was found that the long-term field exposure yielded weaker insulator deterioration than the salt-fog chamber ageing [15], suggesting that long-term salt-fog aging may have a serious impact on the properties loss of HTV. The salt-fog aging phenomenon under corona treatment is more obvious than that without energy [16]. More hydroxylation induced by corona was found in a cold-fog condition, which may cause a hydrophobicity loss of composite insulators [17]. In addition, some detection techniques have been applied to study and evaluate the performance of silicone rubber or composite insulators. For instance, the flashover voltage, or breakdown strength, can reflect the macroscopic electrical insulation property of a material. Static contact angle can tell us something about a material’s hydrophobicity. Fourier infrared spectrum (FTIR) and scanning electron microscopy (SEM) are often used to study the microstructure of silicone rubber. These methods are often used alone or together to study the properties of the material [18,19,20,21,22].

In this paper, the aging experiment of HTV SR samples were conducted in a salt-fog environment with DC energized voltage, and then the electrical strength and physicochemical performances of aged and fresh samples were measured and discussed to study the impact of salt-fog on SR. The variation of DC flashover characteristics was studied. The hydrophobicity change was studied from the results of static contact angles. The microstructure of the sample surface was also observed using the scanning electron microscope. The change of molecular chain bonds was analyzed according to the Fourier infrared spectrum of the samples. The dielectric parameters were proposed to evaluate the insulation performance of HTV silicone rubber. All the research results are discussed to study the effect of salt-fog in the coastal areas on silicone rubber composite insulators from some aspects.

## 2. Materials and Methods

### 2.1. Samples

**Figure 1:**
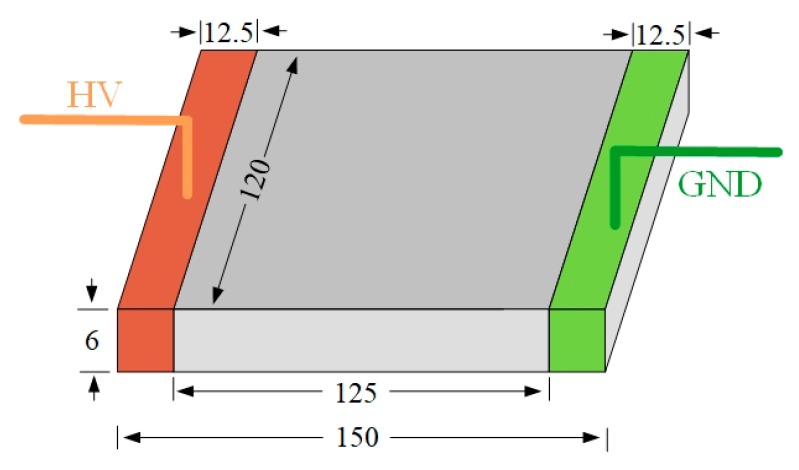
Tested samples (unit: mm).

The silicone rubber samples used in this paper were provided by a Chinese composite insulator manufacturer, and the composition of the silicone rubber samples is the same as that of the composite insulator operating on the transmission line. The main component of this HTV silicone rubber is polymethyl ethylene, and a certain proportion of reinforcing agents (silica and silicate) and flame retardants (aluminum hydroxide) were added. In addition, the samples were a sheet-like shape and the size was 150 mm × 120 mm × 6 mm, shown in Figure 1. Two 12.5 mm electrodes were mounted on each end of the sample during the test process, so the leakage distance of the sample surface was 125 mm.

### 2.2. Experimental Setup

**Figure 2:**
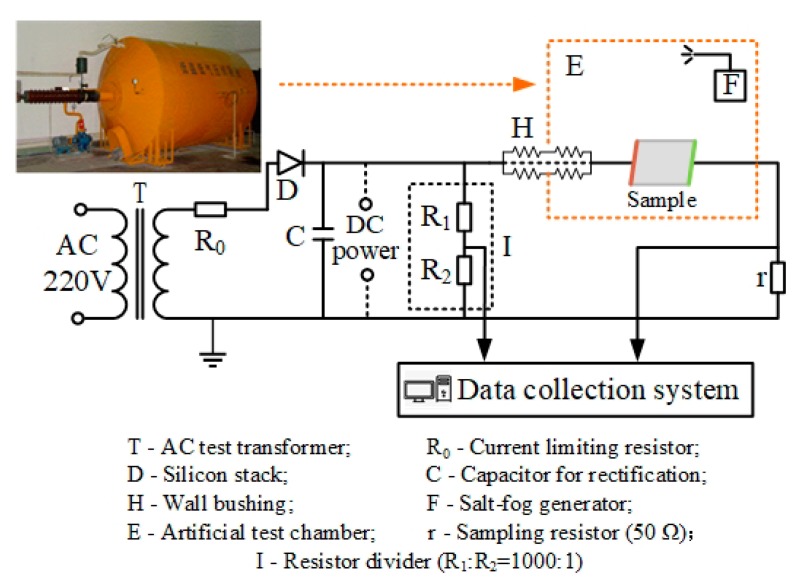
Schematic diagram of DC test circuit.

The aging and flashover tests were carried out in an artificial climate chamber, which had a cylindrical structure and its length was 3.8 m and diameter was 2.0 m. During the test, the salt-fog environment was simulated by generating ultrasonic water mist into the test chamber. The generator can produce salt-fog particles with an amount rate of 2.5 kg/h and these salt water particles had an average size about 1~10 μm. The power supply used in the test was provided by a 150 kV/6 A AC transformer whose maximum short-circuit current was 30 A, meeting the requirements of IEC for the pollution test power supply [23]. The schematic diagram of test circuit is shown in Figure 2, the DC voltage in the test was positive and the meanings of symbols are illustrated in the figure.

### 2.3. Aging and Flashover Test Method with DC Energized

(1) Preparation: Before the aging and flashover test, we carefully cleaned SR samples with absolute ethanol and deionized water and then put them into a dust-free box for 24 h to ensure that the samples’ surfaces had good hydrophobicity [5,21,24,25,26]. According to the relevant regulations of IEC and Chinese standards about the creepage ratio of the light and medium polluted areas in China (35 mm/kV and 40 mm/kV), the applied voltage (*U_a_*) was determined to be 3.6 kV and 3.2 kV since the leakage distance of samples was 125 mm [27,28]. In addition, 1.6 kV and 0 kV (i.e., unenergized) were also selected in order to analyze the influence of applied voltage value.

(2) Aging process: The spray device was first activated, and then a predetermined voltage was applied to the sample to initiate pressure aging for different duration times *t* (2 h, 4 h, 6 h, and 8 h) in the salt-fog environment with different water conductivity rates (γ_20_) of 100 μS/cm, 1000 μS/cm, 3000 μS/cm, and 5000 μS/cm, respectively (all corrected to 20 °C). The temperature of the climate chamber was set at 10 °C. The leakage current waveform was recorded during the period, and the following parameters were calculated based on the current data.

(1) Maximum pulse (*Imax*): The maximum value of the leakage current in a certain period of test time.

(2) Discharge amount (*Q*): The total discharge in a certain period of test time, which is calculated as

(1) $$Q=\int_{0}^{\tau }\left|i(t)\right|dt$$

where *Q* is the discharge amount (C), *i*(*t*) is the leakage current (A), *τ* is the length of time (s).

(3) Electrical strength test: DC flashover voltage was used to characterize the electrical strength of HTV SR in the salt-fog environment. An even-rising voltage method was adopted to find the critical point of electrical strength—we increased the applied voltage uniformly and the flashover voltage was recorded once the flashover occurred. Flashover tests were carried out on 3–4 samples and 4–5 times per sample under the same salt-fog condition. The average flashover voltage *U_f_* and the relative deviation error (σ) were calculated as:

(2) $$U_{f}=\frac{\sum_{i=1}^{N}U_{i}}{N}$$

(3) $$\sigma =\sqrt{\left(\sum_{i=1}^{N}{\left(U_{i}-U_{f}\right)}^{2})/(N-1\right)}/U_{f}\times 100\%$$

where *U_i_* is single flashover voltage, *N* is the total number of valid flashover events for each test condition with different salt-fog conductivity or a different duration time.

### 2.4. Physicochemical Performances Test Methods

The following material properties were tested on samples subjected to an 8 h electrification test in a salt-fog environment, as well as a fresh sample.

#### 2.4.1. Hydrophobicity

A 30 mm × 30 mm tiny sample was cut from the SR sample for the hydrophobicity test. In this paper, we used the static contact angle (CA) to evaluate the hydrophobicity of HTV SR after salt-fog treatment. The measuring device was a DropMeter A-100P video optical contact angle measuring instrument produced by Ouyi, Ningbo, China. Static contact angle measurements were made at nine different positions on the surface of the sample and the results were averaged.

#### 2.4.2. Scanning Electron Microscopy (SEM)

SEM tests were carried out on 1 mm × 2 mm × 3 mm samples that were subjected to a salt-fog experiment with DC energized. The device was a scanning electron microscope manufactured by FEI, Hillsboro, USA with an adjustable magnification of 80–150,000.

#### 2.4.3. Fourier Transform Infrared Spectroscopy (FTIR)

An ALPHA Fourier infrared spectrometer manufactured by Bruker, Karlsruhe, Germany was used for the FTIR test to study the molecular chain bond conditions of samples. In this paper, FTIR was performed on the position where the sample surface was severely burned by arc, and the measurement range was 400 cm^−1^ to 4000 cm^−1^.

#### 2.4.4. Dielectric Parameters

A tiny 30 mm × 30 mm sample of SR was taken for broadband dielectric spectrum scanning to obtain the dielectric parameters of SR sample at different frequencies by an Alpha-A concept 80 wide-band dielectric spectrum analyzer, which was made by Novocontrol Technologies of Montabaur, Germany. The measurement was carried out in a constant temperature box of 25 °C and the frequency was set from 10^−1^ Hz to 10^6^ Hz.

## 3. Results and Discussion

### 3.1. Electrical Strength under Salt-Fog with DC Energized

#### 3.1.1. Fog Water Conductivity

The electrical strength of silicone rubber was investigated according to the aforementioned test procedure. The DC flashover voltage results of samples with different fog water conductivity rates and different duration times are shown in Table 1, the applied voltage was 3.2 kV.

It can be concluded from Table 1 that the fog water conductivity rate has a significant effect on flashover voltage reduction. For instance, for samples energized for 8 h, *U_f_* were 21.3 kV, 11.4 kV, 8.7 kV, and 7.4 kV when γ_20_ was 100 μS/cm, 1000 μS/cm, 3000 μS/cm, and 5000 μS/cm, respectively, which indicates that the *U_f_* decreases by 46.48%, 59.15%, and 65.26%. The reason may be that with the increase of fog water conductivity, the deposition of conductive substances on the silicone rubber surface increases, causing the arc to burn more intensely, then resulting in a decrease in the flashover voltage.

Numerous studies have found that the relationship between the DC pollution flashover voltage and *SDD* can be expressed as follows [29,30,31]:

(4) $$U_{f}=A\cdot SDD^{-n}$$

where *A* is the coefficient related to the insulator material, structure, and power type; *SDD* is the salt deposit density (mg/cm^2^), *n* is the effect coefficient of *SDD* on flashover voltage, while the relationship between *SDD* and γ_20_ can be expressed as [32]:

(5) $$SDD={\left(5.7\gamma_{20}\right)}^{1.03}\cdot (V/S)$$

where γ_20_ is the volume conductivity at the temperature of 20 °C (S/m), *V* is the volume of suspension (cm^3^), *S* is the area of sample surface (cm^2^).

For the salt-fog experiment, the volume of salt-fog generated by spray system and the sample surface area are both constant in a certain period of time. Therefore, referring to Equation (4), the relationship between the flashover voltage *U_f_* and γ_20_ in the salt-fog environment can be expressed as follows:

(6) $$\begin{array}{ll} U_{f} & =A\cdot {\left({\left(5.7\gamma_{20}\right)}^{1.03}\cdot (V/S)\right)}^{-n}\\ & =(A\cdot {\left(6\cdot (V/S)\right)}^{-n})\cdot {\left(\gamma_{20}\right)}^{-1.03n}\\ & =B\cdot \gamma_{20}{}^{-m} \end{array}$$

where *B* is the coefficient related to the silicone rubber material, structure, and power type; γ_20_ is the fog water conductivity (μS/cm); *m* is the effect coefficient of γ_20_ on flashover voltage.

**Figure 3:**
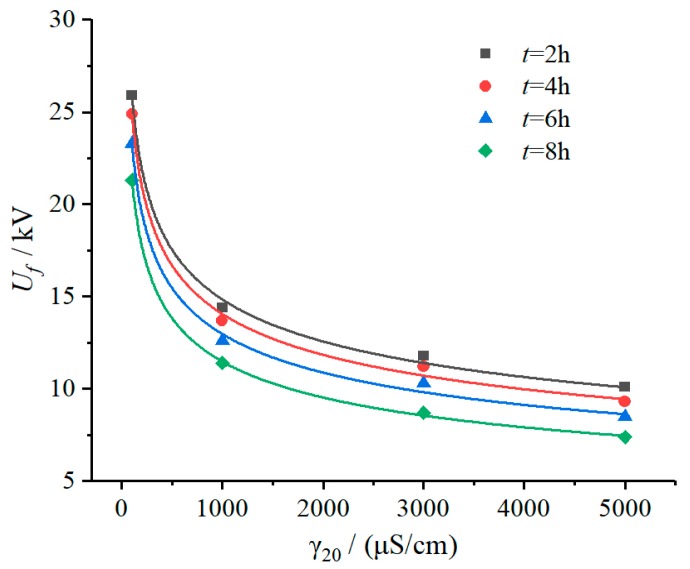
Relationship between *U_f_* and γ_20_ (DC 3.2 kV).

Fitting the test results in Table 1 with Equation (6), the fitted curves and the value of parameters *B* and *m* are shown in Figure 3 and Table 2. It can be seen from the fitting results that the DC flashover voltage of SR samples energized in a salt-fog environment for a period of time decreases exponentially with the increase of fog conductivity.

Table 3 shows the variation of discharge characteristics in different salt-fog conductivity conditions when the applied voltage was 3.2 kV and the duration time was 8 h.

From the leakage current data, it can be seen that during a complete aging test, the maximum pulse (*Imax*), and discharge amount (*Q*) of the leakage current on the sample surface increased significantly with the increase of fog water conductivity. For example, when γ_20_ was 1000 μS/cm, during the test, *Imax* was 0.989 mA and *Q* was 0.136 C, while when γ_20_ increased to 5000 μS/cm, *Imax* was 7.836 mA and *Q* was 5.793 C, which increased by 6.92 times and 41.60 times, respectively, indicating that the salt-fog environment with higher fog water conductivity leads to a more intense discharge.

#### 3.1.2. Duration Time of Applied Voltage

**Figure 4:**
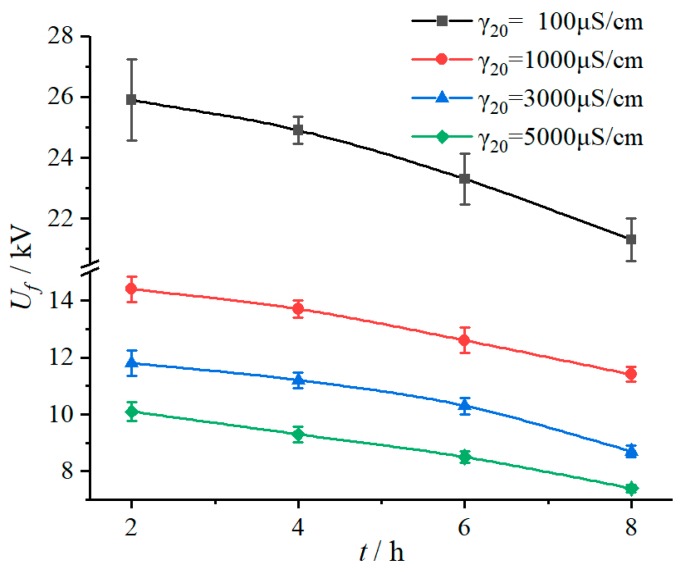
Relationship between *U_f_* and *t* (DC 3.2 kV).

According to the test results in Table 1, under the same fog water conductivity environment, flashover voltage (*U_f_*) declined with increase of duration time of applied voltage (*t*), as shown in Figure 4. In addition, it can be seen from the fitting results in Table 2 that with the extension of duration time, the influence coefficient characteristic exponent m increased. For example, when the duration time increased from 2 h to 4 h, 6 h, and 8 h, the *m* value increased by 2.87%, 5.41%, and 11.58%, respectively, which gives a sign that the fog water conductivity has a greater influence on flashover voltage drop with the duration time increases.

**Figure 5:**
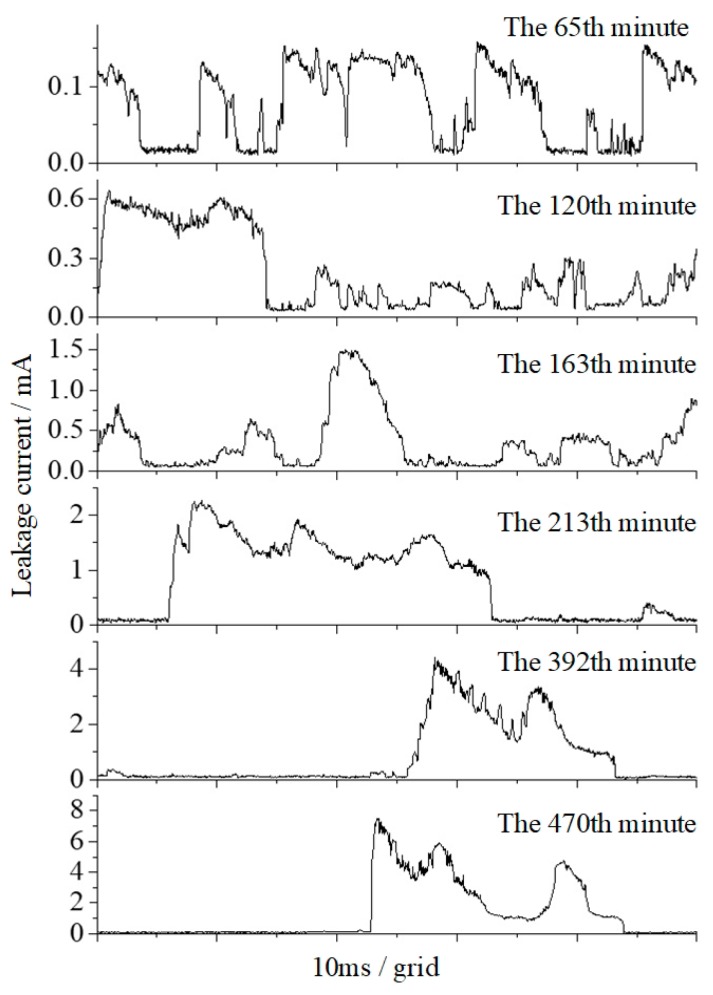
The typical waveform of leakage current (DC 3.2 kV, γ_20_ = 5000 μS/cm).

Table 4 shows the specific values of the leakage current characteristics of different time periods during the 8 h test in the salt-fog condition, γ_20_ is 5000 μS/cm and *U_a_* is 3.2 kV. The current waveform also indicates that the discharge degree was gradually getting stronger, as shown in Figure 5. To be specific, in the first two hours of the test, the maximum pulse amplitude and accumulated discharge amount were 0.682 mA and 0.476 C, which increased to 7.836 mA and 2.646 C in the last two hours, increasing by 10.49 times and 4.56 times, respectively.

#### 3.1.3. Applied Voltage

**Figure 6:**
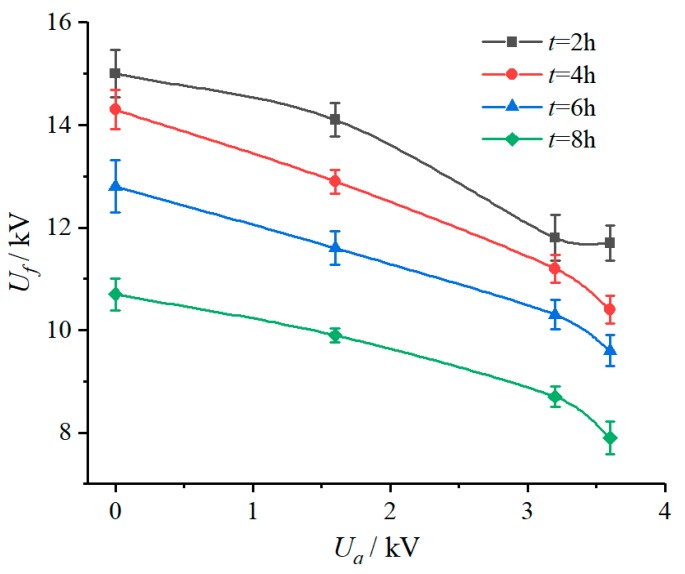
Relationship between *U_f_* and *U_a_* (γ_20_ = 3000 μS/cm).

The DC flashover voltage test results of the samples under different applied voltage conditions are shown in Table 5 and Figure 6. It can be seen that whether the sample was energized in the salt-fog environment and the applied voltage had an influence on the DC flashover voltage. To be specific, in the salt-fog environment with γ_20_ of 3000 μS/cm, compared with 10.7 kV of the unenergized sample, the *U_f_* of samples energized with 1.6 kV, 3.2 kV, and 3.6 kV were 9.9 kV, 8.7 kV, and 7.9 kV, reducing by 7.48%, 18.69%, and 26.17%, respectively. This indicates that in the salt-fog environment, the aging of SR was aggravated by the increase of electric field stress, the hydrophobicity was reduced or even lost, and the insulation performance deteriorated, resulting in a decrease of electrical strength.

Similarly, the leakage current flowing through the surface of the sample increased as the applied voltage increased, as shown in Table 6. In the salt-fog condition with a 3000 μS/cm fog water conductivity rate, the maximum leakage current of the sample with a voltage of 3.6 kV increased by 3.20 times more than that with 1.6 kV, and the cumulative discharge increased by 5.15 times, indicating a more severe discharge under a higher applied voltage.

### 3.2. Physicochemical Performances under Salt-Fog with DC Energized

#### 3.2.1. Hydrophobicity

**Figure 7:**
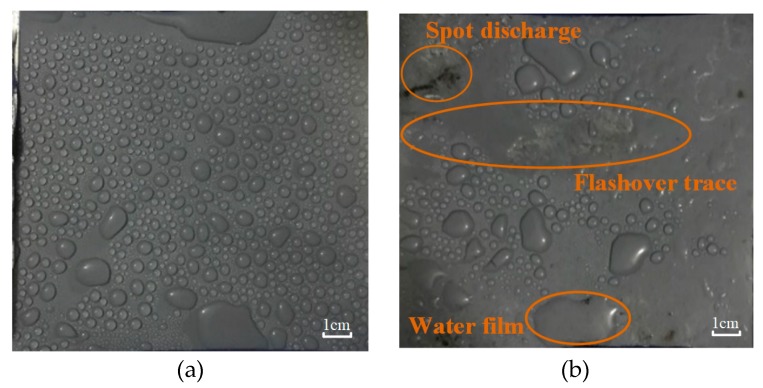
Surface states of the samples after salt-fog treatment. (**a**) Unenergized (0 kV), (**b**) energized (DC 3.2 kV).

Silicone rubber material is widely used in power systems because of its good hydrophobicity. At present, researchers generally believe that materials with a larger static contact angle have better hydrophobicity. However, in our experiment we observed that the static contact angle of SR samples operating in the salt-fog environment with DC energized had decreased. Figure 7 shows the surface states of energized and unenergized samples under the same fog water conductivity rate (γ_20_ = 1000 μS/cm) for the same time (*t* = 8 h).

It can be seen from Figure 7 that the water droplets on the surface of the unenergized sample were distributed evenly and independently, and only small pieces of water film existed in the edge area; while for the energized sample there were dispersed water films and dry-bands on the surface, indicating a loss of hydrophobicity. In addition, in the connection area between water films, traces of arc burning can be clearly seen, indicating that in areas with uneven distribution of water droplets, the electric field was distorted and produced partial discharge, which destroyed the molecular structure and degraded the hydrophobicity of materials.

**Figure 8:**
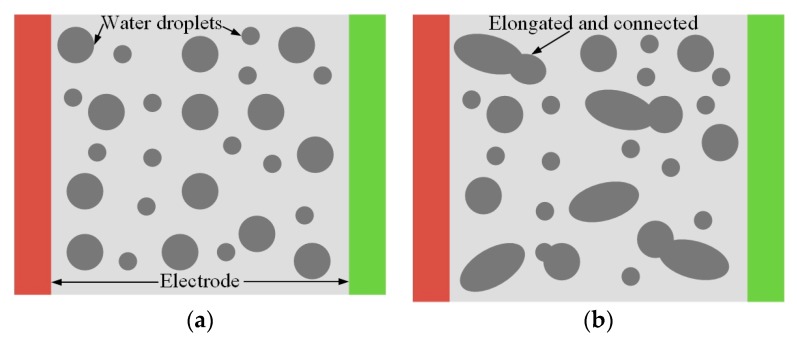

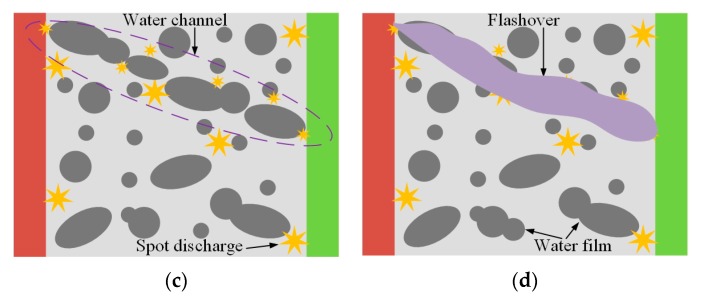
Development of flashover of HTV SR sample in salt-fog environment. (**a**) Water drops deposit, (**b**) water drops deformation, (**c**) partial discharge, (**d**) flashover.

Figure 8 shows the flashover process of the HTV SR sample in the salt-fog environment. (a) Water droplets eventually deposited on the sample surface, distributing evenly and independently with a good hydrophobicity of silicone rubber. At this point, the silicone rubber samples were covered by a high resistive layer, which was scattered with conducting water droplets; (b) the increase of droplet density reduced the distances among the droplets. The DC electric field flattened and elongated the droplets to connect with each other randomly; (c) water film and channel reduced the distance between the electrodes, which led to an increase in the electric field between adjacent wet regions and spot discharges, which consumed the polymer surface around the droplets and destroyed hydrophobicity, eventually causing an elongation of water channel and further spot discharge at the end of channel; (d) increase of the water channel’s length sorts the samples by a conductive, electrolytic path, through which the arc traveled on the surface, finally causing flashover [33,34].

**Figure 9:**
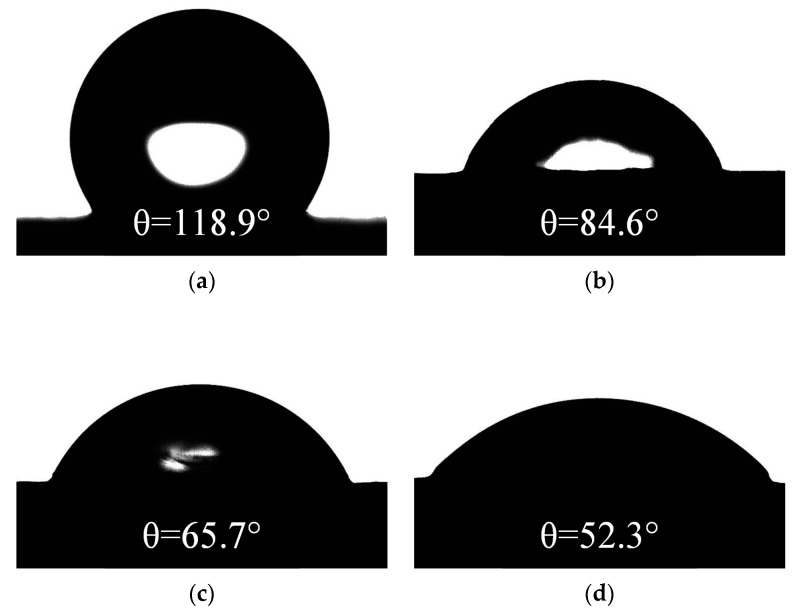
Static contact angles at different applied voltages (γ_20_ = 1000 μS/cm, *t* = 8 h). (**a**) DC 0 kV, (**b**) DC 1.6 kV, (**c**) DC 3.2 kV, (**d**) DC 3.6 kV.

Further, the samples subjected to the 8 h electrification test under different DC voltages were used to measure the static contact angle to study the degradation of the hydrophobicity, as shown in Figure 9.

After the salt-fog experiment, it was found that the decrease of the static contact angle (*CA*) of the SR sample was related to the applied voltage *U_a_*. The higher the value of the *U_a_*, the greater the decrease. For example, the *CA* values of the samples were 84.6°, 65.7°, and 52.3° when *U_a_* were 1.6 kV, 3.2 kV, and 3.6 kV, decreased by 28.85%, 44.74%, and 56.01%, respectively, compared with the unenergized sample (118.9°), which indicates that the hydrophobicity of SR samples has deteriorated.

The degradation of hydrophobicity was essentially caused by the destruction of the molecular structure of the SR material. The higher the applied voltage value, the greater the electric field strength, resulting in more intense discharge. Electrothermal action destroyed the molecular structure of the sample surface, resulting in a decrease in hydrophobic groups and an increase in precipitation of hydrophilic groups and inorganic substances, making the static contact angle smaller, this issue will be discussed further below.

#### 3.2.2. SEM

**Figure 10:**
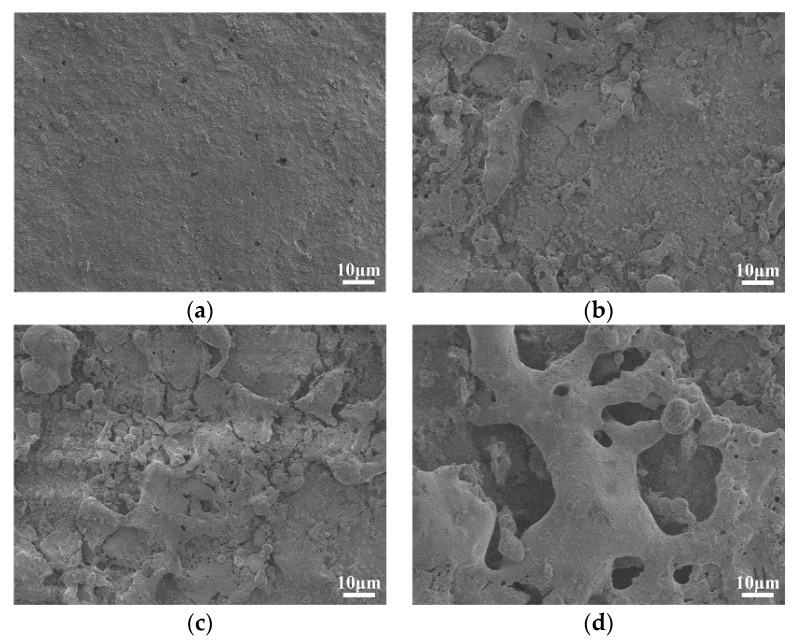
SEM images at different applied voltage (γ_20_ = 1000 μS/cm, *t* = 8 h, 1000×). (**a**) DC 0 kV, (**b**) DC 1.6 kV, (**c**) DC 3.2 kV, (**d**) DC 3.6 kV.

The scanning electron microscope images show the micromorphology of the material surface, which can intuitively reflect the changes of the silicon rubber material surface in the salt-fog environment. Figure 10 shows the SEM images of tested samples under different applied voltages for the 8 h test, γ_20_ is 1000 μS/cm.

It can be seen from Figure 10 that the surface structure of the fresh SR sample was compact, with good flatness and no obvious impurities and defects. On the other hand, the surface structure of the samples energized with DC voltage in salt-fog environment was obviously degraded. The surfaces of tested samples became rough and porous, and were covered with a blocky or cluster-like powder, and with the increase of applied voltage, the destructive effect became more obvious. Since the water droplets easily collapsed on the rough, uneven surface, the static contact angle of electrification-aged samples decreased. This kind of test image also indicates that degradation such as powdering, cracking, and breakage occurs in SR materials operating in the salt-fog environment. According to our follow-up study, it is estimated that the powders on the sample surface may be small molecule siloxane formed by the cleavage of long chain molecules.

#### 3.2.3. FTIR

The Fourier transform infrared spectroscopy is widely used to study the type and relative content of molecular bonds in polymer materials. The wave numbers corresponding to each typical functional group of SR materials are shown in Table 7. In the spectrogram, the higher the absorption peak height, the more the corresponding functional group content.

**Figure 11:**
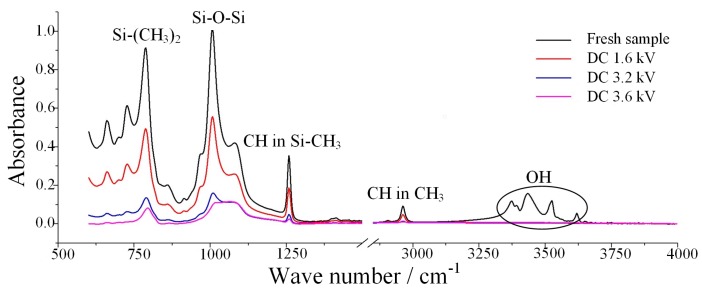
FTIR spectra at different applied voltages (γ_20_ = 1000 μS/cm, *t* = 8 h)

In the present study, by measuring the absorption peak height in a specific band range, the formation, fracture, and recombination of the corresponding chemical bonds were obtained. Thus, the changes in the chemical structure of SR in the salt-fog environment were deduced. The FTIR spectrum for one fresh sample and three energized samples with different DC voltages in a salt-fog condition for 8 h is shown in Figure 11, and Table 8 presents the absorption peak height for corresponding bands.

It can be seen from Figure 11 and Table 8 that the absorption peak height of the main characteristic bands in the infrared spectrum of the sample subjected to the aging test in the salt-fog condition decreased significantly, and the larger the applied voltage value, the more obvious the downward trend. Compared with the fresh sample, the absorption peak height of the energized samples with 3.6 kV had a decrease up to 90%, which indicates that several bonds were seriously broken. The absorption peak height of Si–(CH_3_)_2_ near the band 790–840 cm^−1^ decreased, indicating that the methyl group in the molecular side chain of silicone rubber decreased, and the nonpolar methyl group in the side chain was the structural basis for good hydrophobicity of silicone rubber. Therefore, this change will lead to poor hydrophobicity of the sample, *CA* of water droplets on the silicone rubber surface will become smaller, and adjacent water droplets will be easier to connect with each other, resulting in water film and channel. Similarly, the decrease in the intensity of the adsorption peak at 1000–1100 cm^−1^ confirms that the main Si–O–Si chain of SR molecule was broken. The absorption peak heights in the 1255–1270 cm^−1^ and 2960~2962 cm^−1^ bands also decreased significantly, indicating that the C–H bond within the methyl group also broke, forming free hydrogen. As for the OH bond near 3200–3700 cm^−1^, there were two factors affecting the peak height of it. On the one hand, the free hydrogen element generated by the C–H bond cleavage underwent a hydroxylation reaction with the silicon–oxygen bond to form a new silanol, resulting in an increase in the OH absorption peak. On the other hand, the alumina trihydrate (ATH, a filler material used to inhibit the discharge of SR) [35], decomposed into alumina under the electrothermal action, resulting in a decrease in the OH absorption peak. According to the measurement results, it is obvious that the latter factor played a stronger role, indicating that a large amount of inorganic flame retardants was consumed by the arc burning during the aging test. Therefore, with the gradual consumption of ATH, the arc resistance of the SR material gradually decreased, making the surface of the sample more susceptible to partial discharge, which eventually leads to a reduction in the flashover voltage.

#### 3.2.4. Dielectric Parameter

**Figure 12:**
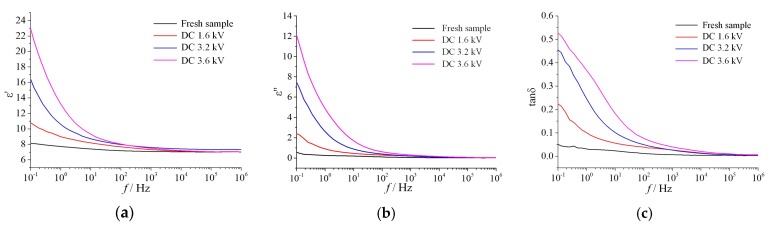
The dielectric parameters at different applied voltage (γ_20_ = 1000 μS/cm, *t* = 8 h). (**a**) Real part, (**b**) imaginary part, (**c**) dielectric loss factor.

Dielectric parameters are the basic physical properties of dielectric, which can characterize the polarization and dielectric loss of materials. In this section, the complex permittivity (including real part ε′ and imaginary part ε′′) and dielectric loss tangent (tanδ) of the SR applied by different applied voltages were obtained, as shown in Figure 12. The specific dielectric parameter values of the samples at three typical frequency points of 10^−1^ Hz, 50 Hz, and 10^4^ Hz are shown in Table 9.

It can be concluded from Figure 12 and Table 9 that both the ε and tanδ of energized samples were obviously higher than the fresh sample. In the salt-fog experiment process, the arc cauterization caused holes and cracks on the samples’ surfaces, which leads to a loose and porous surface, so the moisture is more easily able to invade the sample. Thus, the intrusion of moisture increases the polarization and dielectric loss of SR material. Finally, the insulation performance of the material deteriorates and the flashover voltage decreases.

## 4. Conclusions

In this paper, a series of performances of HTV SR samples in a salt-fog environment with DC energized voltage were studied. It was found that the electrical strength as well as physicochemical performances of the material were degraded to some degree.

The discharge of the SR surface was more intense when samples were in a high fog water conductivity condition. The DC flashover voltage decreased with the increase of fog water conductivity in a negative exponential pattern, decreased with the increase of duration time, and decreased with the increase of applied voltage.

The hydrophobicity of tested samples was reduced as CA became smaller. Water film and dry-band appeared on the surface of silicone rubber.

SEM images showed that there was a change of surface morphology from compact to rough and porous. Some physical defects and cluster particles were observed, indicating that arc discharge has a destructive effect on SR.

Analysis of FTIR spectra showed a hydroxylation and decomposition of ATH, this can bring down the arc resistance of silicone rubber and makes it easier for water droplets to collapse and accumulate on the sample surface, resulting in a decrease in electrical strength.

The dielectric parameter of the tested material also increased significantly, indicating that the insulation properties of the tested sample deteriorated.

In general, in the high-conductivity salt-fog environment, the energized silicone rubber samples are prone to sustained partial discharge, which will damage the material itself and degrade the performance. The properties discussed above all indicate the degradation of silicone rubber materials from one certain aspect. In fact, the changes in various properties will affect each other. For example, according to the analysis of FTIR, the chemical bonds of the silicone rubber were broken under the bombardment of high-energy particles, thereby generating small-molecule siloxanes and inorganic particles, which increased the roughness of the sample surface and reduced the hydrophobicity of the material. These degradations in performance ultimately affect the electrical strength of the material, making flashovers more likely to occur.

## Figures and Tables

**Table 1:** DC flashover results at different fog water conductivity rates (DC 3.2 kV).

*t* (h)	γ_20_ (μS/cm)							
	100		1000		3000		5000	
	*U_f_* (kV)	σ (%)	*U_f_* (kV)	σ (%)	*U_f_* (kV)	σ (%)	*U_f_* (kV)	σ (%)
2	25.9	5.2	14.4	3.1	11.8	3.8	10.1	3.4
4	24.9	1.8	13.7	2.2	11.2	2.4	9.3	2.9
6	23.3	3.6	12.6	3.5	10.3	2.8	8.5	2.4
8	21.3	3.3	11.4	2.3	8.7	2.3	7.4	1.7

**Table 2:** Fitting results of flashover voltage.

*t* (h)	*B*	*m*
2	77.99	0.2401
4	77.49	0.2470
6	74.54	0.2531
8	73.12	0.2679

**Table 3:** Characteristic quantity of leakage current at different fog water conductivity rates (DC 3.2 kV, *t* = 8 h).

γ_20_ (μS/cm)	100	1000	3000	5000
*Q*/C	0.136	1.594	2.639	5.793
*I_max_*/mA	0.989	1.972	2.832	7.836

**Table 4:** Specific values of leakage currents at different time periods (DC 3.2 kV, γ_20_ = 5000 μS/cm).

*t* (h)	*Q* (C)	*I_max_* (mA)
0~2	0.476	0.682
2~4	0.937	2.368
4~6	1.734	3.732
6~8	2.646	7.836

**Table 5:** DC flashover results at different applied voltage (γ_20_ = 3000 μS/cm).

*t* (h)	*U_a_* (kV)							
	0		1.6		3.2		3.6	
	*U_f_* (kV)	σ (%)	*U_f_* (kV)	σ (%)	*U_f_* (kV)	σ (%)	*U_f_* (kV)	σ (%)
2	15.0	3.1	14.1	2.3	11.8	3.8	11.7	2.9
4	14.3	2.7	12.9	1.8	11.2	2.4	10.4	2.6
6	12.8	4.0	11.6	2.8	10.3	2.8	9.6	3.1
8	10.7	2.9	9.9	1.4	8.7	2.3	7.9	4.0

**Table 6:** Characteristic quantities of leakage current at different applied voltage (γ_20_ = 3000 μS/cm, *t* = 8 h).

*U_a_* (kV)	*Q* (C)	*I_max_* (mA)
1.6	0.498	0.697
3.2	2.639	2.832
3.6	3.062	2.927

**Table 7:** FTIR absorption bands of silicone rubber.

Wavenumber (cm^−1^)	Functional Groups	Characteristics
790~840	Si–(CH_3_)_2_	CH_3_ stretching vibration
1000~1100	Si–O–Si	Si–O stretching vibration
1255~1270	CH in Si–CH_3_	CH_3_ symmetrical deformation vibration
2960~2962	CH in CH_3_	C–H stretching vibration
3200~3700	OH	Condensation OH stretching vibration

**Table 8:** The absorption peak height of bands in the FTIR spectrum.

Samples	Wavenumber (cm^−1^)					
	790~840	1000~1100	1255~1270	1412	2960~2962	3200~3700
Fresh sample	0.914	1.000	0.354	0.030	0.092	0.156
DC 1.6 kV	0.493	0.556	0.186	0.017	0.048	0.007
DC 3.2 kV	0.135	0.160	0.048	0.005	0.014	0.006
DC 3.6 kV	0.080	0.110	0.024	0.003	0.009	0.004

**Table 9:** Variation of dielectric parameters at different applied voltage (γ_20_ = 1000 μS/cm, *t* = 8 h).

Samples	10^−1^ Hz			50 Hz			10^4^ Hz		
	ε′	ε″	tanδ	ε′	ε″	tanδ	ε′	ε″	tanδ
Fresh sample	8.09	0.58	0.071	7.23	0.13	0.018	7.03	0.03	0.004
DC 1.6 kV	10.81	2.42	0.224	7.77	0.34	0.044	7.10	0.07	0.010
DC 3.2 kV	16.42	7.45	0.454	8.12	0.48	0.059	7.18	0.09	0.012
DC 3.6 kV	22.94	12.08	0.527	8.25	0.76	0.092	7.41	0.14	0.019
